# Study of insulin resistance in relation to serum IGF-I levels in subjects with different degrees of glucose tolerance

**DOI:** 10.4103/0973-3930.43100

**Published:** 2008

**Authors:** Srinivas Martha, Narayana Pantam, Surender Thungathurthi, Vummentala L. N. Rao, Krishna Devarakonda

**Affiliations:** University College of Pharmaceutical Sciences, Kakatiya University, Warangal - 506 009, Andhra Pradesh, India; 1 Department of General Medicine, Mahatma Gandhi Memorial Hospital, Warangal - 506 002, Andhra Pradesh, India

**Keywords:** Diabetes mellitus, insulin like growth factor-I (IGF-I), insulin resistance syndrome

## Abstract

**AIM:**

We studied the correlations between fasting and post-lunch serum IGF-I concentrations, and insulin resistance and insulin sensitivity in subjects with various degrees of glucose tolerance.

**MATERIALS AND METHODS:**

A total of 12 nondiabetic subjects, 09 subjects with impaired glucose tolerance (IGT) and 18 patients with newly diagnosed type-2 diabetes of either sex (mean age, 46 years) were recruited. None of the participants received any drug treatment at the commencement of the study. Fasting as well as post-lunch blood samples were collected from all the subjects and anthropometric and biochemical parameters were analyzed.

**RESULTS:**

Fasting serum IGF-I concentrations were negatively correlated with fasting serum glucose, insulin, C-peptide, triglycerides, total LDL and VLDL cholesterol, homeostatic model assessment of insulin resistance (HOMA-IR), and age. Fasting serum IGF-I concentrations were positively correlated with fasting blood HDL cholesterol and homeostatic model assessment of insulin sensitivity (HOMA-S) in only diabetic subjects. Post-lunch serum IGF-I concentrations were positively correlated with HDL and LDL cholesterol. Correlations with HOMA-S with these metabolic anthropometric variables were of similar magnitude and direction as that of IGF-I concentrations. IGF-I concentrations were significantly lower in the subjects with World Health Organization-defined metabolic syndrome compared with the subjects without metabolic syndrome (*P* < 0.0001).

**CONCLUSIONS:**

Our data indicate that IGF-I could be a useful marker in the insulin resistance syndrome. The post-lunch low-IGF-I levels help in better identification of subjects at risk for type-2 diabetes mellitus and cardiovascular disease.

## Introduction

Insulin resistance is a chronic metabolic syndrome characterized by a cluster of abnormalities including altered glucose tolerance, visceral adiposity, hypertension, low HDL cholesterol and high triglyceride levels. It is linked with so many cardiovascular diseases.[1–2] There is an increasing evidence in the last few years suggesting that IGF-I may have a role in both glucose homeostasis and atherosclerotic cardiovascular disease. Animals with absence of liver specific IGF-I gene are characterized by hyperinsulinemia and skeletal muscle insulin resistance.[3,4] These animals when treated with recombinant IGF-I showed a reduction in insulin levels and an increase in insulin sensitivity.[3] Clinical studies performed in patients with extreme insulin resistance and in patients with both types of diabetes have shown that recombinant IGF-I administration significantly lowered blood glucose and increased insulin sensitivity.[5–8] A recent study concluded that low concentrations of IGF-I in the blood circulation was increasingly associated with increased risk of developing type-2 diabetes.[9] In nondiabetic subjects, the low levels of IGF-I in circulation have been associated with angiographically reported coronary artery diseases.[10,11] In patients with effort angina pectoris and angiographically normal epicardial coronary arteries (cardiac syndrome-X), low levels of IGF-I have been observed.[12] Individuals with low levels of IGF-I have increased risk of developing ischemic heart disease compared to individuals with normal levels of IGF-I.[13] Nondiabetic patients succumbing to an acute myocardial infarction had significantly lower IGF-I levels than survivors.[14] In view of these findings, a close link between metabolic syndrome-X and insulin resistance, diabetes, and atherosclerotic coronary artery diseases might be attributed to the low levels of circulating IGF-I, which in turn affects insulin sensitivity. In the present study, we have evaluated the correlations between IGF-I concentrations and insulin sensitivity in both fasting and post-lunch serum samples using anthropometric and biochemical variables in subjects with different degrees of glucose tolerance.

## Materials and Methods

The study included 12 nondiabetic subjects, 09 subjects with impaired glucose tolerance (IGT), and 18 patients with newly diagnosed type-2 diabetes. The study was conducted at the Department of General Medicine, Mahatma Gandhi Memorial Hospital, Warangal, AP, India, from March 2006 to June 2007. All the subjects were attending general health checkup at our out-patient department. Subjects were excluded if they had chronic gastrointestinal diseases associated with malabsorption, chronic pancreatitis, history of any malignant disease, history of alcohol abuse, kidney or liver failure, and drug treatment that could modify the glucose homeostasis. IGT and type-2 diabetes mellitus were diagnosed as per the American Diabetes Association criteria based on fasting and post-lunch blood glucose levels (IGT-FBS, 110–126; PLBS, 130–140; DM-FBS, 140–200; and PLBS, >200 mg/dl).[15]

### Ethics

The study was approved by Institutional Ethics Committee (Kakatiya Medical College, Warangal) and informed consent was obtained from each subject according to the principles of the declaration of Helsinki.

### Biochemical assays

Fasting and post-lunch blood glucose, total and HDL cholesterol, and triglycerides were measured by enzymatic methods (Excel Diagnostics Private Limited, Hyderabad, India). Fasting and post-lunch serum insulin concentrations were determined by enzyme-linked immunosorbent assay (ELISA) (Mercodia AB., Sweden). Fasting and post-lunch serum C-peptide concentrations were determined by ELISA (Biomerica Inc., USA). Fasting and post-lunch serum IGF-I concentrations were determined by ELISA (Mediagnost., Germany). Fasting and post-lunch serum VLDL and LDL cholesterol concentrations were determined by using the formula: VLDL cholesterol = triglycerides/5; and LDL cholesterol = total cholesterol – (HDL + VLDL cholesterol).[16] Insulin resistance was assessed by using the previously validated homeostasis model assessment for insulin resistance, calculated from the fasting insulin and glucose concentrations according to the formula: HOMA-IR = [(insulin × glucose)/22.5].[17] Similarly, insulin sensitivity was assessed by using the previously validated homeostasis model assessment for insulin sensitivity, calculated from the fasting insulin and glucose concentrations according to the formula: HOMA-S = 1 / [(insulin X glucose) X 22.5].[18]

### Statistical analysis

All variables are expressed as mean ± SD. Group differences of continuous variables were compared using ANOVA followed by Newman Keuls test. Relationships between variables were determined by Pearson's correlation coefficient. For all analyses, a *P* value > 0.05 was considered to be statistically significant. All analyses were performed using GraphPad Prism 4 Demo software.

## Results

Anthropometric and biochemical characteristics of the study subjects are shown in Table 1. Subjects with IGT and newly diagnosed type-2 diabetes were similar in age and had significantly higher fasting and post-lunch blood glucose (*P* < 0.0001, *P* < 0.0001, respectively), and not significantly higher fasting and post-lunch serum insulin, C-peptide, abnormal lipid profile, and HOMA-IR levels compared with nondiabetic subjects. HDL cholesterol was not significantly lower in subjects with IGT and diabetes compared with nondiabetic subjects. Interestingly, HOMA-S was not significantly lower in subjects with IGT and type-2 diabetes (*P <* 0.323). Fasting and postlunch serum IGF-I concentrations were significantly lower in subjects with IGT as well as type-2 diabetes (*P <* 0.0001, *P <* 0.007, respectively).

**Table 1:** Anthropometric and biochemical characteristics of the study subjects

Parameter	Normal subjects		IGT subjects		Newly detected DM subjects		*P*-value	
				
	Fasting	Post lunch	Fasting	Post lunch	Fasting	Post lunch	Fasting	Post lunch
Sex (Male/Female)	6/6	6/6	7/2	7/2	17/1	17/1	-	-
Age (years)	45.58 ± 8.56	45.58 ± 8.56	44.89 ± 4.73	44.89 ± 4.73	47.83 ± 9.06	47.83 ± 9.06	0.614	-
Glucose (mg%)	76.83 ± 14.49	106.25 ± 14.0	119.67 ± 5.6	215.56 ± 25.6	209.39 ± 64.1	314.61 ± 70.5	0.0001	0.0001
Insulin (μU/ml)	4.39 ± 2.68	16.04 ± 12.87	5.72 ± 3.10	22.48 ± 27.18	3.72 ± 3.14	7.2 ± 8.45	0.276	0.059
C-Peptide (ng/ml)	1.20 ± 0.99	5.48 ± 3.00	4.08 ± 6.55	7.97 ± 4.47	2.12 ± 4.08	5.56 ± 4.07	0.303	0.264
IGF-I (ng/ml)	144.99 ± 6.70	150.60 ± 11.6	99.69 ± 15.02	113.78 ± 27.48	87.59 ± 29.12	108.93 ± 45.7	0.0001	0.007
Triglycerides (mg%)	145.96 ± 36.1	203.03 ± 90.7	202.69 ± 199	206.73 ± 145	249.83 ± 171	251.85 ± 154	0.20	0.56
Total cholesterol (mg%)	255.56 ± 47.1	266.67 ± 57.0	279.01 ± 60.2	324.69 ± 91.5	295.68 ± 74.8	327.16 ± 88.2	0.25	0.118
HDL cholesterol (mg%)	68.38 ± 15.11	59.31 ± 11.48	65.35 ± 4.1	52.61 ± 5.96	65.03 ± 7.20	58.98 ± 7.1	0.64	0.14
LDL cholesterol (mg%)	157.98 ± 42.7	166.75 ± 56.8	173.1 ± 65.3	230.7 ± 83.2	187.4 ± 60.2	217.8 ± 82.8	0.38	0.11
VLDL cholesterol (mg%)	29.19 ± 7.23	40.61 ± 18.1	40.54 ± 39.9	41.35 ± 29	49.97 ± 34.2	50.37 ± 30.83	0.20	0.56
HOMA -IR	0.89 ± 0.63		1.70 ± 0.91		1.89 ± 1.62		0.102	-
HOMA-S	6.13 ± 15.69		1.00 ± 0.96		1.70 ± 2.75		0.323	-

Data are mean ± SD, group differences of variables were compared using ONE WAY ANOVA

Univariate correlations between both serum IGF-I concentrations and established components of the insulin resistance syndrome were assessed for the study subjects [Table 2a, 2b, and 2c]. In all the subjects fasting and post-lunch serum IGF-I concentrations were negatively correlated with age, blood glucose, insulin, C-peptide, triglycerides, and total lipid profile with an exception of HDL cholesterol and insulin resistance expressed as HOMA-IR. Fasting serum IGF-I concentrations were positively correlated with HDL cholesterol and insulin sensitivity (HOMA-S). Post-lunch serum IGF-I concentrations were positively correlated with LDL cholesterol in addition to HDL cholesterol. Correlations for HOMA-S with these metabolic variables were of a similar degree and in the same direction as that serum IGF-I concentrations. HOMA-S was negatively correlated with age, glucose, insulin, C-peptide, triglycerides, total cholesterol, and HOMA-IR. In contrast, it was positively correlated with HDL cholesterol and serum IGF-I.

**Table 2a:** Univariate correlations with IGF-I and HOMA-S concentrations of normal subjects

	Normal subjects					
	
	Fasting				Post lunch	
		
	IGF-I		HOMA-S		IGF-I	
			
	r	*P*	r	*P*	r	*P*
Age	0.79	0.009*	−0.27	0.37	0.01	0.95
Glucose	0.18	0.56	−0.40	0.18	−0.46	0.12
Insulin	0.21	0.51	−0.55	0.05*	0.07	0.81
C-Peptide	−0.36	0.23	−0.22	0.48	0.05	0.86
Triglycerides	−0.55	0.06	0.28	0.36	−0.13	0.67
Total cholesterol	0.62	0.03*	−0.19	0.53	0.46	0.13
HDL cholesterol	0.76	0.003*	−0.14	0.65	0.08	0.79
LDL cholesterol	0.5	0.09	−0.21	0.49	0.49	0.11
VLDL cholesterol	−0.55	0.06	0.28	0.36	−0.13	0.67
HOMA-IR	0.16	0.61	−0.49	0.1
HOMA-S	−0.36	0.24	-	-	-	-
IGF-I	-	-	-0.36	0.24	-	-

^r^Pearson's correlation coefficient

* *P*-value <0.05 is considered statistically significant

**Table 2b:** Univariate correlations with IGF-I and HOMA-S concentrations of IGT subjects

	IGT subjects					
	
	Fasting				Post lunch	
		
	IGF-I		HOMA-S		IGF-I	
			
	r	*P*	r	*P*	r	*P*
Age	0.12	0.74	−0.55	0.12	−0.06	0.86
Glucose	−0.03	0.92	−0.17	0.64	−0.31	0.41
Insulin	0.14	0.70	−0.87	0.002*	−0.17	0.65
C-Peptide	−0.55	0.12	−0.24	0.53	−0.22	0.56
Triglycerides	−0.44	0.22	−0.23	0.53	−0.47	0.19
Total cholesterol	−0.26	0.49	−0.02	0.95	−0.01	0.96
HDL cholesterol	−0.41	0.26	0.04	0.91	0.001	0.99
LDL cholesterol	0.05	0.88	0.12	0.75	0.14	0.70
VLDL cholesterol	−0.44	0.22	−0.23	0.53	−0.47	0.19
HOMA-IR	0.12	0.75	−0.88	0.001*	-	-
HOMA-S	−0.27	0.47	-	-	-	-
IGF-I	-	-	−0.27	0.47	-	-

rPearson's correlation coefficient

* *P*-value <0.05 is considered statistically significant

**Table 2c:** Univariate correlations with IGF-I and HOMA-S concentrations of newly detected diabetic subjects

	Newly detected diabetic subjects					
	
	Fasting				Post lunch	
		
	IGF-I		HOMA-S		IGF-I	
			
	r	*P*	r	*P*	r	*P*
Age	−0.32	0.19	−0.25	0.29	−0.35	0.14
Glucose	0.31	0.19	−0.19	0.43	−0.09	0.71
Insulin	−0.40	0.09	−0.51	0.02*	−0.15	0.54
C-Peptide	−0.24	0.33	0.009	0.97	0.33	0.18
Triglycerides	−0.08	0.72	−0.02	0.91	−0.04	0.84
Total cholesterol	0.48	0.04*	0.03	0.87	0.19	0.42
HDL cholesterol	0.07	0.75	−0.11	0.64	0.19	0.43
LDL cholesterol	0.51	0.03*	0.05	0.83	0.21	0.39
VLDL cholesterol	−0.08	0.72	−0.02	0.91	−0.04	0.84
HOMA-IR	−0.36	0.14	−0.52	0.02*	-	-
HOMA-S	0.26	0.29	-	-	-	-
IGF-I	-	-	0.26	0.29	-	-

rPearson's correlation coefficient

* *P* value <0.05 is considered statistically significant

However, the correlations between fasting serum IGF-I concentrations and established components of the insulin resistance syndrome including age and HDL cholesterol were significant in nondiabetic subjects, but not in IGT and newly detected type-II diabetic patients; and postlunch serum IGF-I did not show significant correlation with age and HDL cholesterol. The correlations between fasting and postlunch serum IGF-I concentrations and established components of the insulin resistance syndrome including blood glucose, insulin, C-peptide, triglycerides, VLDL cholesterol, HOMA-IR, and HOMA-S were not significant in all subjects. The correlations between fasting serum IGF-I concentrations and total and LDL cholesterol were significant in nondiabetic subjects and patients with newly detected type-2 diabetes, but not in IGT subjects and all the groups of subjects of post-lunch serum samples. The correlations between serum insulin sensitivity calculated from HOMA-S and serum insulin resistance estimated from HOMA-IR were statistically significant in all subjects, while serum blood glucose, C-peptide, triglycerides, total lipid profile, and IGF-I were not. The correlations of serum IGF-I and HOMA-S with all established components of insulin resistance syndrome are shown in the Table 2.

## Discussion

Serum IGF-I levels were positively correlated with insulin sensitivity and HDL cholesterol and negatively correlated with all components of insulin resistance syndrome. These relations of HOMA-S were similar in degree and directions to those observed with serum IGF-I levels. Furthermore, low levels of serum IGF-I were significantly related with the metabolic syndrome according to the World Health Organization.[19] However, our findings are consistent with the animal studies demonstrating low insulin sensitivity in mice with liver specific deletion of the IGF-I gene that is reversed by treatment with recombinant human IGF-I.[3,4] Raised blood pressure and slightly enhanced plasma insulin concentrations have been reported in mice with a mutant IGF-I allele causing a marked decrease in circulating IGF-I levels.[20]

The present investigation was limited by the fact that the analyses were based on total IGF-I rather than the biologically active free IGF-I. Moreover, the analyses of total IGF-I is very high, it is also possible that the nutritional and genetic factors influence the actual levels of circulating IGF-I. In this regard, a polymorphism in the promoter region of the IGF-I gene was associated with low levels of IGF-I and increased risk of type-2 diabetes mellitus.[21] In addition to this, IGF-I binding proteins (IGFBPs) were not measured, which play an important role in regulating the bioavailability of IGF-I. Among IGFBPs, IGFBP-3 is very important since it binds more than 95% of IGF-I in blood circulation.[22]

IGF-I has hypoglycemic effects and enhances insulin sensitivity in both experimental and human subjects, which is due to its type-1 receptors and/or hybrid insulin/IGF-I receptors.[23] However, it is not clear how low IGF-I levels may induce insulin insensitivity. The insulin receptors are widely distributed in human tissues[24] and behave as IGF-I homoreceptor rather than insulin homoreceptor or some intermediate of the two receptors.[25] It has been proposed that the hybrid insulin/IGF-I receptors would reduce insulin sensitivity in target tissues of insulin action leading to insulin resistance. Because of this fact, in patients with insulinoma, a condition characterized by marked hyperinsulinemia, downregulation of insulin receptors induced by elevated plasma insulin was associated with increased abundance of hybrid insulin/IGF-I receptors.[26]

Most of the circulating IGF-I is derived from its synthesis in the liver, regulated by growth hormone (GH), insulin, and nutritional intake.[27] Low levels of circulating IGF-I may be because of inadequate negative feedback at the level of the hypothalamus and/or pituitary; thus, resulting in GH hypersecretion and a decrease in insulin sensitivity. In support of this possibility, it has been shown that mice with liver specific deletion of the IGF-I gene exhibited elevated GH levels, which were associated with insulin resistance and impaired activation of early signaling events in response to insulin.[3] It has been demonstrated that blocking of GH action in mice with liver specific deletion of the IGF-I gene by crossing them with mice over-expressing a mutant form of GH, which prevents GH activation of its receptor, results in improved insulin sensitivity. Considering these results together, it was suggested that GH hypersecretion may be a major determinant of insulin resistance in subjects with low-plasma-IGF-I concentrations.

In conclusion, the present study indicates that serum IGF-I concentrations, particularly low levels, may be a useful marker for identifying subjects at risk of developing type-2 diabetes mellitus and possible cardiovascular complications. Especially, post-lunch serum IGF-I levels may be important in identifying the onset of type-2 diabetes mellitus. The difference between fasting IGF-I and post-lunch IGF-I concentrations in IGT and type-2 diabetes mellitus are directly proportionate, probably indicating nonutilization of IGF-I in post-lunch state. Further prospective and clinical intervention studies in large number of subjects are necessary to definitively prove this hypothesis.

## References

[1] Ginsberg HN (2000). Insulin resistance and cardiovascular disease. J Clin Invest.

[2] Reaven GM (1988). Banting lecture 1988: Role of insulin resistance in human disease. Diabetes.

[3] Yakar S, Liu JL, Fernandez AM, Wu Y, Schally AV, Frystyk J (2001). Liver-specific IGF-1gene deletion leads to muscle insulin insensitivity. Diabetes.

[4] Sjogren K, Wallenius K, Liu JL, Bohlooly- YM, Pacini G, Svensson L (2001). Liver-derived IGF-I is of importancefor normal carbohydrate andlipid metabolism. Diabetes.

[5] Moses AC, Young SC, Morrow LA, O'Brien M, Clemmons DR (1996). Recombinant human insulin-like growth factor I increases insulin sensitivity and improves glycemic control in type II diabetes. Diabetes.

[6] Morrow LA, O'Brien MB, Moller DE, Flier JS, Moses AC (1994). Recombinant human insulin-like growth factor-I therapy improves glycemic control and insulin action in the type A syndrome of severe insulin resistance. J Clin Endocrinol Metab.

[7] Carroll PV, Umpleby M, Ward GS, Imuere S, Alexander E, Dunger D (1997). rhIGF-I administration reduces insulin requirements, decreases growth hormone secretion, and improves the lipid profile in adults with IDDM. Diabetes.

[8] Thrailkill KM, Quattrin T, Baker L, Kuntze JE, Compton PG, Martha PM (1999). Cotherapy with recombinant human insulin-like growth factor I and insulin improves glycemic control in type 1 diabetes: RhIGF-I in IDDM Study Group. Diabetes Care.

[9] Sandhu MS, Heald AH, Gibson JM, Cruickshank JK, Dunger DB, Wareham NJ (2002). Circulating concentrations of insulin like growth factor-I and development of glucose intolerance: a prospective observational study. Lancet.

[10] Spallarossa P, Brunelli C, Minuto F, Caruso D, Battistini M, Caponnetto S (1996). Insulin-like growth factor-I and angiographically documented coronary artery disease. Am J Cardiol.

[11] Janssen JA, Stolk RP, Pols HA, Grobbee DE, Lamberts SW (1998). Serum total IGF-I, free IGF-I, and IGFBP-1 levels in an elderly population: Relation to cardiovascular risk factors and disease. Arterioscler Thromb Vasc Biol.

[12] Conti E, Andreotti F, Sestito A, Riccardi P, Menini E, Crea F (2002). Reduced levels of insulin-like growth factor-1 in patients with angina pectoris, positive exercise stress test, and angiographically normal epicardial coronary arteries. Am J Cardiol.

[13] Juul A, Scheike T, Davidsen M, Gyllenborg J, Jørgensen T (2002). Low serum insulin like growth factor I is associated with increased risk of ischemic heart disease: A population-based case-control study. Circulation.

[14] Friberg L, Werner S, Eggertsen G, Ahnve S (2000). Growth hormone and insulin-like growth factor-I in acute myocardial infarction. Eur Heart J.

[15] Dorin R (1997). The human genome project: impact on the prevalence of diabetes.

[16] Irene G, Tsimihodimos V, Theodosios DF, Vasilios GS, Eleni TB (2006). LDL cholesterol estimation in patients with the metabolic syndrome. Lipids Health Dis.

[17] Matthews DR, Hosker JP, Rudenski AS, Naylor BA, Treacher DF, Turner RC (1985). Homeostasis model assessment: Insulin resistance and β-cell function from fasting glucose and insulin concentrations in man. Diabetologia.

[18] Alberti KG, Zimmet PZ (1998). Definition, diagnosis and classification of diabetes mellitus and its complications, Part 1: Diagnosis and classification of diabetes mellitus provisional report of a WHO consultation. Diabet Med.

[19] Lembo G, Rockman HA, Hunter JJ, Steinmetz H, Koch WJ, Ma L (1996). Elevated blood pressure and enhanced myocardial contractility in mice with severe IGF-1 deficiency. J Clin Invest.

[20] Vaessen N, Heutink P, Janssen JA, Wittman JC, Testers L, Hofman A (2001). A polymorphism in the gene for IGF-I: Functional properties and risk for type 2 diabetes and myocardial infarction. Diabetes.

[21] Jones JI, Clemmons DR (1995). Insulin-like growth factors and their binding proteins. Endocr Rev.

[22] Sesti G, Federici M, Lauro D, Sbraccia P, Lauro R (2001). Molecular mechanism of insulin resistance in type 2 diabetes mellitus: Role of the insulin receptor variant forms. Diabetes Metab Res Rev.

[23] Federici M, Porzio O, Zucaro L, Fusco A, Borboni P, Lauro D (1997). Distribution of insulin/insulin-like growth factor-I hybrid receptors in human tissues. Mol Cell Endocrinol.

[24] Seely BL, Reichart DR, Takata Y, Yip C, Olefsky JM (1995). A functional assessment of insulin/insulin-like growth factor-I hybrid receptors. Endocrinology.

[25] Federici M, Lauro D, D'Adamo M, Giovannone B, Porzio O, Mellozzi M (1998). Expression of insulin/IGF-I hybrid receptors is increased in skeletal muscle of patients with chronic primary hyperinsulinemia. Diabetes.

[26] Holt RI, Simpson HL, Sonksen PH (2003). The role of the growth hormone insulin-like growth factor axis in glucose homeostasis. Diabetes Med.

[27] Yakar S, Setser J, Zhao H, Stannard B, Haluzik M, Glatt V (2004). Inhibition of growth hormone action improves insulin sensitivity in liver IGF-1-deficient mice. J Clin Invest.

